# Costs in the Year Following Deceased Donor Kidney Transplantation: Relationships With Renal Function and Graft Failure

**DOI:** 10.3389/ti.2022.10422

**Published:** 2022-05-27

**Authors:** Matthew Cooper, Mark Schnitzler, Chanigan Nilubol, Weiying Wang, Zheng Wu, Robert J. Nordyke

**Affiliations:** ^1^ Medstar Georgetown Transplant Institute, Washington, DC, United States; ^2^ School of Medicine, Saint Louis University, St. Louis, MO, United States; ^3^ Genesis Research, Hoboken, NJ, United States; ^4^ Beta6 Consulting Group, Los Angeles, CA, United States

**Keywords:** kidney transplant, graft failure, estimated glomerular filtration rate, graft function, cost

## Abstract

Relationships between renal function and medical costs for deceased donor kidney transplant recipients are not fully quantified post-transplant. We describe these relationships with renal function measured by estimated glomerular filtration rate (eGFR) and graft failure. The United States Renal Data System identified adults receiving single-organ deceased donor kidneys 2012–2015. Inpatient, outpatient, other facility costs and eGFRs at discharge, 6 and 12 months were included. A time-history of costs was constructed for graft failures and monthly costs in the first year post-transplant were compared to those without failure. The cohort of 24,021 deceased donor recipients had a 2.4% graft failure rate in the first year. Total medical costs exhibit strong trends with eGFR. Recipients with 6-month eGFRs of 30–59 ml/min/1.73m^2^ have total costs 48% lower than those <30 ml/min/1.73m^2^. For recipients with graft failure monthly costs begin to rise 3–4 months prior to failure, with incremental costs of over $38,000 during the month of failure. Mean annual total incremental costs of graft failure are over $150,000. Total costs post-transplant are strongly correlated with eGFR. Graft failure in the first year is an expensive, months-long process. Further reductions in early graft failures could yield significant human and economic benefits.

## Graphical Abstract

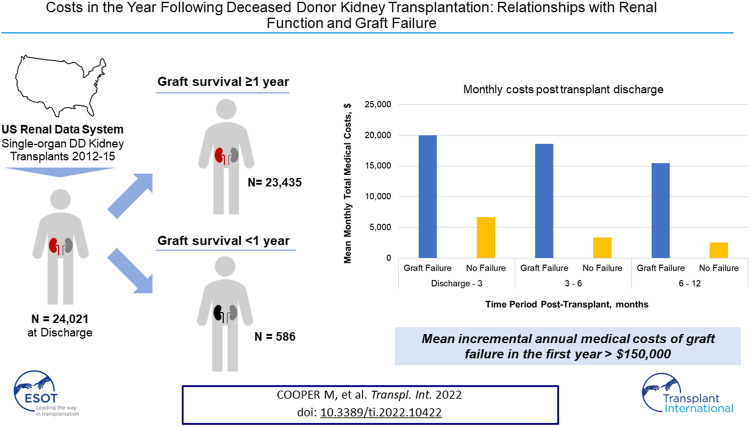

## Introduction

Kidney transplantation is the preferred treatment for end-stage kidney disease (ESKD) with greater patient survival than dialysis as well as far lower annual costs (1–3) Early achievement and maintenance of good renal function in the first year post-transplant has been shown to be associated with both improved graft survival and lower costs in subsequent time periods. Kidney function within the first year of transplantation has been consistently identified as a key factor that affects longer term graft survival in observational and experimental settings (4–17) Further, clinical and administrative claims data from the USRDS have shown that 12-month estimated glomerular filtration rate (eGFR) is strongly associated with costs in the second and third years post-transplant among those surviving to at least 1 year post-transplant (18, 19) Additionally, eGFR at 6 months post-transplant has also demonstrated a strong association with hospitalizations in the following 12 month period (20).

This body of evidence has helped inform patient management practices aimed at maintaining renal function in the first months post-transplant to help realize the recent gains in long-term human and economic benefits (21, 22) Chief among these efforts has been improved immunosuppressant management to minimize acute rejection, which are costly events impairing short-term renal function and which increase long-term failure risks (23).

Graft failure is relatively rare in the first year post-transplant. However it is a very costly clinical event by itself in addition to the subsequent return to dialysis and, perhaps, later transplant. Overall, the economic costs of failure in terms comparing the costs of those with functioning grafts versus those on maintenance dialysis have been well-characterized over the years (24–27) For example in the US, reference data from the USRDS Annual Reports demonstrates that when a kidney transplant fails, the incremental cost to Medicare is approximately $95,000 in the first full-year of failure and annual costs for continuing maintenance dialysis patients are over 3 times greater than for those grafts for more than 1 year (28) Factors likely driving these costs include increased healthcare utilization peri-failure as well as the increased costs of the return to dialysis and, less frequently, rapid re-transplantation. While mean renal function in a cohort of transplant recipients stabilizes by 3 months post-transplant, there remains substantial variation in individual patient eGFR outcomes in the first year (14, 29–33).

Thus, the time-dependence of the relationships between graft function and medical costs in the first year post-transplant are not well studied: what are the short-term economic implications of patient management practices that improve renal function in the first year post-transplant? Our aims were to describe relationships between total medical costs in the first year following deceased donor kidney transplant with overall renal function as measured by: 1) eGFR at different time points for recipients with graft survival of at least 12 months, and; 2) graft failure for those with survival ≤12 months. We used the USRDS claims dataset to assess these real-world economic relationships within 1 year post kidney transplantation.

## Materials and Methods

### Data Sources

A retrospective cohort of single-organ, deceased donor kidney transplantation recipients was identified using the United States Renal Data System (USRDS) database from 1 January 2012 to 31 December 2015. One year follow-up was allowed to 31 December 2016. The USRDS database is a joint effort of the National Institute of Diabetes and Digestive and Kidney Disease (NIDDK) and the Centers for Medicare and Medicaid Services (CMS) that tracks many descriptive elements for all patients in the US with end-stage kidney disease (ESKD). USRDS registries integrate information from the Organ Procurement and Transplantation Network (OPTN), CMS, and Medicare billing claims records (2) These elements are linked with a unique encrypted patient identifier, permitting investigators to combine patient-specific information from multiple tables without revealing patient identity. Data provided by USRDS are deidentified thus this analysis was exempt from IRB review.

### Study Population

This study population included adult (18+) deceased donor kidney transplant patients with Medicare as their primary payer at time of first transplantation from 2012 to 2015. If patients had multiple kidney transplant procedures in that time period, the first was included. Patients with other organ transplants prior to the index transplantation were excluded from this study.

Patients without serum creatinine measures at discharge, at 180 and 365 days post-transplantation were excluded from this analysis (Table 1). Patients who died with a functioning graft during the first year were excluded from the analysis following their death.

**TABLE 1:** Recipient demographic and clinical characteristics.

Category	All patients		Graft failure^a^		No graft failure^a^	
Overall, N (% of total)	24,021	100%	586	2.4%	23,435	97.6%
Age Group, N (%)						
<30	1,213	5.1%	33	5.6%	1,180	5.0%
30–45	4,488	18.7%	89	15.2%	4,399	18.8%
45–59	8,811	36.7%	214	36.5%	8,597	36.7%
60–74	8,859	36.9%	222	37.9%	8,637	36.9%
≥75	650	2.7%	28	4.8%	622	2.7%
Age						
Mean (SD)	54.0	13.09	55.2	13.54	54.0	13.08
Gender						
Male	14,705	61.2%	359	61.3%	14,346	61.2%
Female	9,316	38.8%	227	38.7%	9,089	38.8%
Race						
Black	8,735	36.4%	239	40.8%	8,496	36.3%
Non-Black	15,286	63.6%	347	59.2%	14,939	63.8%
BMI [kg/m^2^]						
Mean (SD)	28.6	5.45	29.3	5.75	28.6	5.44
Cause of ESRD						
Polycystic kidney disease	1,607	6.7%	19	3.2%	1,588	6.8%
Diabetes	7,291	30.4%	180	30.7%	7,111	30.3%
Glomerulonephritis	5,144	21.4%	142	24.2%	5,002	21.3%
Hypertension	6,580	27.4%	169	28.8%	6,411	27.4%
Other	3,399	14.2%	76	13.0%	3,323	14.2%
Year of Transplant						
2012	5,774	24.0%	146	24.9%	5,628	24.0%
2013	5,949	24.8%	149	25.4%	5,800	24.8%
2014	5,993	25.0%	140	23.9%	5,853	25.0%
2015	6,305	26.3%	151	25.8%	6,154	26.3%

a Within 12 months post-transplant.

### Patient Baseline Characteristics

The following patient demographics and clinical variables were assessed in this study (Table 1): age at transplantation, gender, assigned race (Black vs. Non-Black), body mass index (BMI), and cause of ESKD. Baseline patient demographics were assessed for the entire sample population and stratified further for patients who experienced graft failure and those who did not during the first 12 months post-transplant.

### eGFR and Cost Variable Definitions

Costs were derived from Parts A and B claims including inpatient, emergency, outpatient, and skilled nursing facility costs. Costs were included following discharge from the initial transplant hospitalization. Note that, for patients with graft failure, dialysis costs are not reported separately by USRDS and are largely incorporated into the Outpatient cost category. eGFRs were available at discharge, and at 6 and 12 months. Thus, eGFR:Cost relationships are described for several month-based time periods post-discharge: 0–3, 3–6, and 6–12 months. Serum creatinine measures were included in they were within 2 weeks of the discharge, 6, or 12 month time periods. All costs were adjusted to 2019 USD. In this study, eGFR was assessed using the Chronic Kidney Disease Epidemiology Collaboration (CKD-EPI) equation, using serum creatinine (34, 35).

### Statistical Analysis

Descriptive statistics were used to describe the baseline demographic and clinical variables. Continuous variables were described with mean and standard deviation (SD). Categorical variables were described with counts and percentage. Data were missing for calculation of BMI for a few patients (146/24,021, 0.6%) and these missing values were imputed using the mean from patients with complete data. Both descriptive and multivariate generalized linear models with standard gamma distribution and log link function were used to define the relationship between eGFR measures and total medical costs.

For recipients with graft failure a time-history of medical costs was constructed with failure as the index date (Month 0 in Figure 3). Descriptive analyses were conducted in which monthly costs of those with graft failure in the first year post-transplant were compared to those without failure. Monthly costs for those without graft failure in the first year were a composite measure. This composite was created for each possible month pre- and post-index date by assigning the mean medical cost of non-failures to the month post-transplant in which failures occurred. This matching process appropriately reflects the higher costs experienced by all recipients in the first months post-transplant. This reporting followed the STROBE guidelines.

## Results

### Demographic and Clinical Characteristics

Patients who received a single-organ deceased donor kidney transplant from 2012 to 2015 experienced a 1-year graft failure rate of 2.4% (Table 1). Compared to those without early graft failure, these recipients tended to be slightly older (mean age: 55.2 vs. 54.0 years) and had marginally higher BMI (29.3 vs. 28.6 kg/m^2^). A greater proportion of recipients with early graft failure were Black (40.8% vs. 36.3%) and were less likely to have PKD (3.2% vs. 6.8%) and more likely to have glomerulonephritis (24.2% vs. 21.3%) as cause of ESRD. Among those without graft failure in the first year post-transplant over 96% had serum creatinine measurements at discharge, and at 6 and 12 months post-transplant.

### Overall Medical Costs

**FIGURE 1:**
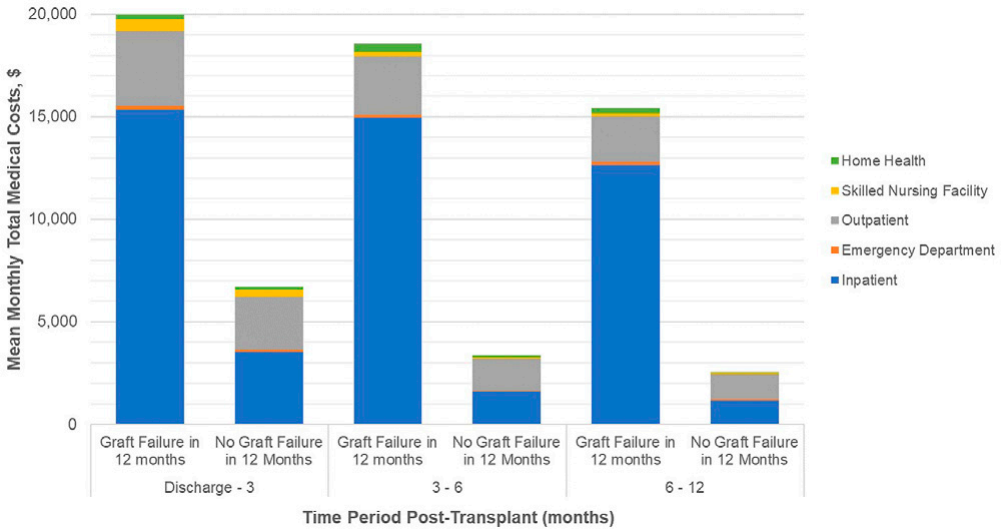
Healthcare costs (PPPM) post transplant discharge date, by time period post-surgical discharge. Footnotes: Mean hospice costs are ≤ $3 per month and are not reported here. Please refer to Supplementary Table S3. All differences in total monthly medical costs between those with and without graft failure in any given time period are significant at P<0.001.

Mean monthly medical costs in the first year post-transplant (Figure 1) are substantially higher for recipients who had graft failure while monthly costs for both groups show similar downward trends over the first year post-transplant. Mean monthly costs in the early time period, discharge to 3 months, are 3.0 times higher for graft failures versus those without failure ($19,992 vs. $6,681). This ratio increases to a factor of 6.0 in the 6–12 month timeframe ($15,436 vs. $2,555).

By treatment setting, inpatient costs are the largest driver of cost differentials between patient groups. In the discharge to 3 month period, mean monthly inpatient costs are 4.4 times higher in the graft failure group ($15,341 vs. $3,520) and 10.9 times higher ($12,637 vs. $1,163) in the 6–12 month period. Outpatient costs are consistently higher for those with graft failure in the first year; during the early period mean monthly medical costs are 42% higher than non-failures ($3,638 vs. $2,562) and, while absolute OP costs decrease overall, the differential rises to 81% higher during the 6–12 month period ($2,218 vs. $1,226). Other medical costs (skilled nursing facility, emergency department, and home health) are small components of total costs but are consistently higher for those with graft failure.

### eGFR and Cost Outcomes

**FIGURE 2:**
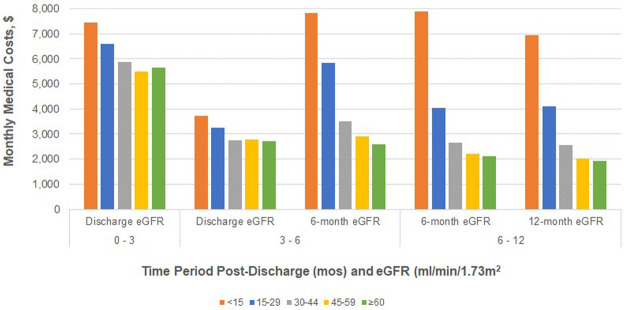
Adjusted total medical costs (PPPM) post-transplant discharge date for patients without graft failure, by eGFR measurement and time period post-surgical discharge.

The relationships between monthly medical costs and eGFR measurements at different timepoints demonstrate consistently strong trends (Figure 2). Both unadjusted and adjusted (Supplementary Table S4) relationships are similar in that eGFR is strongly correlated with medical costs in all time periods (*p* < 0.001 for each trend). The trend in the discharge to 3 month time period appears to be the least strong likely due to high variance among serum creatinine values at discharge (Supplementary Table S2) and continuing variability in renal function soon after transplant. For time periods after 3 months, the relationship between eGFR measurements and mean monthly medical costs are quite similar. Adjusted costs are highest ($7,157 to $7,826 per month) for those with a functioning graft and eGFR<15 ml/min/1.73 m^2^. In the 6–12 month time period, for example, adjusted monthly medical costs are over 40% lower for those with 6-month eGFRs 15–29 ml/min/1.73 m^2^ than for those with 6-month eGFRs <15 ml/min/1.73 m^2^ ($4,546 vs. $7,799) and costs are another 35% lower for 30–44 ml/min/1.73 m^2^ as compared to 15–29 ml/min/1.73m^2^ ($2,919 vs. $4,546). In addition to eGFR measures, age, gender, race, BMI, and cause of ESRD are important determinants of mean monthly costs in most time periods (Supplementary Table S5). The model fit parameters indicate that 12-month eGFR measurements are somewhat more strongly correlated with mean monthly costs in the 6–12 month time period than are 6-month eGFR measurements in the adjusted analyses.

### Early Graft Failure and Cost Outcomes

**FIGURE 3:**
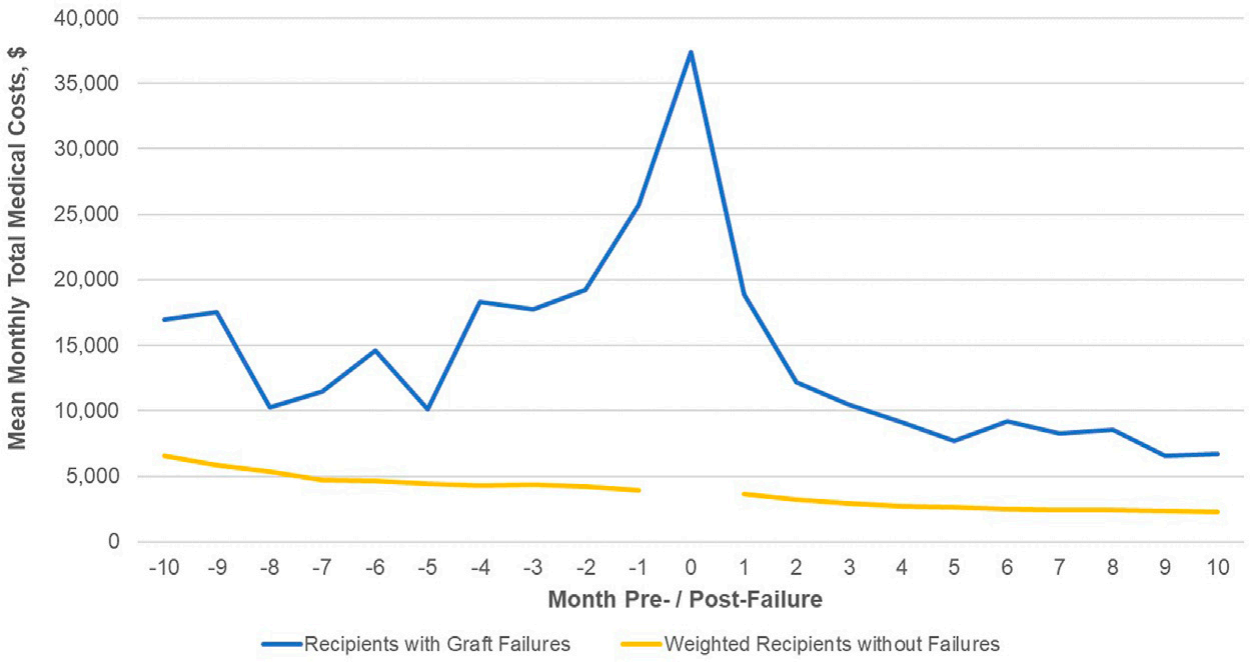
Total medical costs (PPPM) post-transplant discharge date for patients with graft failure. Footnote: No cost data exist for the index month (Month 0) for the comparison group of patients without graft failure.

For recipients with graft failure monthly costs begin to rise 3, 4 months prior to failure, with a spike of over $38,000 during the month of failure (Figure 3 and Supplementary Table S6). Costs appear to stabilize 3, 4 months post-failure suggesting a failure process that is several months long. Mean monthly costs ≥6 months post-failure are about 3 times those for patients without failure. Compared to the monthly costs of patients without graft failure weighted for the month post-transplant, costs for those experiencing graft failure are higher at each month observed. Centering on the median month of failure, 6 months post-transplant, the incremental costs of graft failure are $153,000 in the first year, as compared to those without graft failure.

## Discussion

This analysis of deceased donor recipients with Medicare as primary payer demonstrates the strong relationship between medical costs and graft function in the first year post-transplant. For those recipients without early graft failure, there is a consistent trend toward lower total medical costs among patients with higher eGFR. Among non-failures, adjusted total medical costs are comparable for those with eGFRs above 45 ml/min/1.73 m^2^ and increase exponentially for those with eGFRs below 45 ml/min/1.73 m^2^. These patterns hold from 3 months post-transplant and regardless whether 6-month or 12-month eGFR is used as the reference point, reflecting the relative stability of renal function for the majority of patients without early graft failure. These results confirm that current patient management practices aimed at achieving and maintaining good renal function post-transplant also provide net economic benefits as well. As a contrasting point, current organ allocation/procurement policies have improved broader/more equitable distribution of organs with resulting longer CIT, higher rates of DGF, and higher sCr early post-transplant which, collectively, may tend to reduce eGFR among the cohort of transplant recipients. Our findings therefore suggest there may be an unrecognized economic cost of our constrained organ supply.

Time histories of medical costs for recipients who do experience early graft loss illustrate the high overall costs of failure. These trends also indicate that, from a healthcare utilization standpoint, graft failure is a process that may begin as early as several months prior to the failure event itself and that continues for 2–3 months as patients return to dialysis or retransplantation.

Regardless of treatment setting, mean medical costs are higher for those with early failure than those without. The dominant factor driving the higher total costs is inpatient utilization where costs are a factor of 4x to 10x in the early time period (0–3 months) and later time period (6–12 months), respectively. The peri-failure period is a time of particularly complex and intensive care management. Evidence that there is slightly higher patient mortality risk for each 1 ml/min/1.73 m^2^ higher eGFR at dialysis reinitiation (36, 37) underscores the criticality; declaring failure too early is detrimental to patient outcomes as is waiting too long. Therefore, constant monitoring is required to identify appropriate time to initiate dialysis, with admissions, more biopsies and more frequent OP visits to monitor changes in SCr. In addition, most patients will require care to prepare for dialysis, including evaluation and placement of vascular access. For a relative few, evaluation for pre-emptive re-transplantation requires additional care. Post-failure, nephrectomy may be indicated in many patients to eliminate risks of graft intolerance syndrome as well as graft rupture or hemorrhage. This complex peri-failure care management is also highly-dependent on an individual patient’s history, status, and preferences. That management places a significant burden on patients in direct out-of-pocket costs for care, but also indirect costs of time, travel, and family support. Disadvantaged patients may therefore not receive optimal care, thereby increasing the total cost of care and the time required to return to stable care post-failure. This is an under-studied area. An interesting area of future research would include an analysis of how the relationship between renal function and medical costs as patient management have improved over time.

Important limitations of this study are those typical for retrospective analyses of observational data, including the potential for residual confounding associated with factors not available in the study database. Additionally, given the study inclusion criteria, costs may not be generalizable to all DD recipient populations. For example, we excluded patients without Medicare as primary payer and our results may not apply to patients with employer group health plans. More generally, USRDS data lacks detailed laboratory and histology data that may be appropriate to include in our regression analyses of the eGFR:cost relationship regarding certain data elements and this lack of specificity may be important for prospective study. While additional control variables, if included, might be shown to be statistically significant determinants of first-year costs, our analysis suggests that they would not achieve the dominant role of renal function.

Our findings demonstrate that total medical costs in the first year post-transplant are substantially correlated with renal function, measured both as graft failure and as eGFR among those with surviving grafts at various times post-discharge. These results indicate that continued focus on improving renal function and reducing early graft failures could yield significant human and economic benefits.

## Data Availability

The data analyzed in this study is subject to the following licenses/restrictions: Access to USRDS data requires a formal application and acceptance of a Data Use Agreement. Requests to access these datasets should be directed to USRDS, https://www.usrds.org/for-researchers/standard-analysis-files.

## Abbreviations

BMI, body mass index; CIT, cold ischemia time; CKD-EPI, chronic kidney disease epidemiology collaboration; CMS, centers for medicare and medicaid services; DGF, delayed graft function; eGFR, estimated glomerular filtration ratio; ESKD, end-stage kidney disease; NIDDK, national institute of diabetes and digestive and kidney disease; OPTN, organ procurement and transplantation network; PKD, polycystic kidney disease; USRDS, United States renal data system.
