# Supplementary material for: Costs in the Year Following Deceased Donor Kidney Transplantation: Relationships With Renal Function and Graft Failure
Source: Transpl Int. 2022 May 27;35:10422. doi: 10.3389/ti.2022.10422 (PMC9184448; doi:10.3389/ti.2022.10422)
Supplement: Supplementary file 1 [file DataSheet1.PDF]

SUPPLEMENTAL MATERIAL

Supplemental Table S1: Cohort Attrition. Source: United States Renal Data System, 2012-2016.

	**Criteria (applied sequentially)**	**N**	**%**
Step 1	Patients who had only 1 kidney transplant from 2012 – 2015	67,360	100.0%
Step 2	Patients aged 18 and older at time of transplant	64,487	95.7%
Step 3	Excluding patients with multi-organ transplants	62,372	92.6%
Step 4	Patients with no kidney transplants prior to 2012	54,674	81.2%
Step 5	Patients with deceased donors only	35,623	52.9%
Step 6	Patients with Medicare as primary payer during the date of transplant	24,501	36.4%
Step 7	Patients with at least one serum creatinine levels post-transplant (near discharge, at 6 month, or 12 month)	24,485	36.3%
Step 8	Exclude patients with missing discharge date	24,446	36.3%
Step 9	Exclude patients who had graft failure or died during transplant admission	24,121	35.8%
Step 10	Exclude patients with extreme BMI values (BMI>100 or BMI<6)	24,114	35.8%
Step 11	Exclude patients whose initial hospitalization stay greater than 45 days	24,021	35.7%

Supplemental Table S2: Recipient serum creatinine levels (mg/dl) in first year post-transplant, by failure cohort and time period

Category	All Patients		Graft Failure*		No Graft Failure*	
**Near discharge**						
N (% of sub-group)	24,012	99.9%	586	100.0%	23,426	99.9%
Mean (SD)	3.91	2.99	5.29	3.11	3.88	2.98
**6 months post-transplant**						
N (% of sub-group)	23,172	96.5%	195	33.3%	22,977	98.1%
Mean (SD)	1.43	0.61	3.15	2.17	1.41	0.55
**12 months post-transplant**						
N (% of sub-group)	22,527	93.8%	19	3.2%	22,508	96.0%
Mean (SD)	1.42	0.61	4.95	2.2	1.41	0.6

Supplemental Table S3: Data for Figure 1 – Mean Medical Costs (PPPM) post transplant discharge date, by time period

**Time post-transplant:**	**0-3 mos**		**3-6 mos**		**6-12 mos**	
Graft Failure status within 12 months post-transplant	**Graft Failure**	**No Graft Failure**	**Graft Failure**	**No Graft Failure**	**Graft Failure**	**No Graft Failure**
**Treatment Setting**						
Inpatient	15,341	3,520	14,952	1,581	12,637	1,163
Emergency Dept.	205	129	157	60	168	52
Outpatient	3,638	2,562	2,847	1,554	2,218	1,226
Skilled Nursing	568	345	214	81	141	60
Hospice Costs	0	1	0	2	0	3
Home Health	240	124	403	74	272	52
**Total Medical Cost**	**19,992**	**6,681**	**18,574**	**3,351**	**15,436**	**2,555**

Supplemental Table S4 – Total medical costs (PPPM) post-transplant discharge date for patients without graft failure, by eGFR measurement and time period, Unadjusted and Adjusted

	Time Post-Transplant	0 – 3 mos	3 – 6 mos		6 – 12 mos	
eGFR Category (ml/min/1.73m^2)	*eGFR Measurement*	Discharge eGFR	Discharge eGFR	6-month eGFR	6-month eGFR	12-month eGFR
	**Unadjusted Mean Monthly Medical Costs**					
<15		7,695	3,873	8,137	8,287	7,137
15-29		6,732	3,422	6,153	4,811	4,680
30-44		5,962	2,865	3,668	3,100	3,088
45-59		5,544	2,893	3,021	2,564	2,419
≥60		5,628	2,786	2,650	2,366	2,181
	**Adjusted Mean Monthly Medical Costs**					
<15		7,458	3,725	7,826	7,799	7,157
15-29		6,607	3,254	5,850	4,546	4,481
30-44		5,856	2,739	3,507	2,919	2,942
45-59		5,479	2,776	2,899	2,424	2,315
≥60		5,648	2,723	2,591	2,269	2,103

Supplemental Table S5 – Regression results for Adjusting Monthly Medical Costs (2019 USD) and eGFR. Estimated coefficients from GLM regression reported.

Category	Discharge through 3 month post-transplant		Months 3 through 6 Post-transplant		Months 6 through 12 Post-transplant			
	eGFR near discharge		eGFR at 6 months post-transplant		eGFR at 6 months post-transplant		eGFR at 12 months post-transplant	
	Estimate	P-value	Estimate	P-value	Estimate	P-value	Estimate	P-value
**eGFR (Reference: ≥60)**								
45-<60	**-0.0301**	0.2773	**0.1147**	<.0001	**0.0387**	0.0456	**0.0547**	0.004
30-<45	**0.0368**	0.1541	**0.3059**	<.0001	**0.2189**	<.0001	**0.2877**	<.0001
15->30	**0.1575**	<.0001	**0.8186**	<.0001	**0.6442**	<.0001	**0.7565**	<.0001
<15	**0.2788**	<.0001	**1.1108**	<.0001	**1.3102**	<.0001	**1.2838**	<.0001
**Age (Ref: <30)**								
30 to 44	**-0.005**	0.8863	**-0.0675**	0.0835	**-0.0638**	0.1155	**-0.0607**	0.1255
45 to 59	**0.0863**	0.0114	**0.0451**	0.2318	**0.0555**	0.1576	**0.0398**	0.2991
60 to 74	**0.2314**	<.0001	**0.1305**	0.0006	**0.1401**	0.0004	**0.0922**	0.0173
75+	**0.3137**	<.0001	**0.1366**	0.0224	**0.092**	0.1382	**0.0428**	0.4819
**Gender (Ref: Female)**								
Male	**-0.0673**	<.0001	**-0.0195**	0.2248	**-0.0711**	<.0001	**-0.0773**	<.0001
**Race (Ref: Non-black)**								
Black	**0.1383**	<.0001	**0.0641**	0.0001	**0.0881**	<.0001	**0.0704**	<.0001
**Cause of ESRD (Reference: Other)**								
Polycystic kidney	**-0.2422**	<.0001	**-0.1663**	<.0001	**-0.2867**	<.0001	**-0.2523**	<.0001
Diabetes	**0.0972**	<.0001	**0.1496**	<.0001	**0.1445**	<.0001	**0.1702**	<.0001
Glomerulonephritis	**-0.0739**	0.002	**-0.0902**	0.0007	**-0.1685**	<.0001	**-0.1554**	<.0001
Hypertension	**-0.0905**	0.0001	**-0.0975**	0.0002	**-0.1247**	<.0001	**-0.1076**	<.0001
**Body Mass Index [kg/m2]**								
BMI	**0.0015**	0.2279	**0.0023**	0.1155	**-0.0031**	0.0353	**-0.0032**	0.0271
Model Fit								
AIC	458,274		412,812		401,108		390,499	

Supplemental Table S6 – Data for Figure 3 for those with graft failure.

X Month* pre/post the Graft Failure date	N**	Mean	SD
Prior to graft failure			
Month -10	66	16,946	33,058
Month -9	101	17,549	41,822
Month -8	138	10,251	13,087
Month -7	167	11,465	22,642
Month -6	216	14,603	31,819
Month -5	262	10,155	15,977
Month -4	314	18,306	40,489
Month -3	402	17,766	50,515
Month -2	473	19,230	36,838
Month -1	535	25,754	43,407
Month 0	***586***	***37,416***	***49,105***
Post-graft failure			
Month 1	529	18,878	51,178
Month 2	475	12,211	20,266
Month 3	422	10,484	16,504
Month 4	374	9,161	18,184
Month 5	338	7,681	14,870
Month 6	316	9,221	17,470
Month 7	274	8,306	14,323
Month 8	236	8,585	20,541
Month 9	196	6,555	10,137
Month 10	116	6,721	8,937

* Month 0 was defined as up to 31 days in which a graft failure occurs, +/- 15 days centered on the failure date. Patients were followed up to 12 months post-transplant:

- Month -m (m=-10 to -1): From graft failure date to discharge date for up to 12 months (Month -1(exclude 15 days pre-graft failure date) - Month -12);
- Month m (m=1 to 10): From graft failure date to 12-month post-transplant for up to 12 months (Month 1(exclude 15 days post-graft failure date) - Month 12)"

**N for Month -m (m=-1 to -10) prior to graft failure: number of patients who have graft failure between (m-0.5) months post discharge and 12-month post transplant;
N for Month 0 (graft failure month): number of patients who have graft failure between discharge and 12-month post transplant;
N for Month m (m=1 to 10) post graft failure: number of patients who have graft failure between discharge and (12.5 - m) months post transplant.
